# Targeting miR‐126 disrupts maintenance of myelodysplastic syndrome stem and progenitor cells

**DOI:** 10.1002/ctm2.610

**Published:** 2021-10-14

**Authors:** Huafeng Wang, Jie Sun, Bin Zhang, Dandan Zhao, Hongyan Tong, Herman Wu, Xia Li, Yingwan Luo, Dan Dong, Yiyi Yao, Tinisha McDonald, Anthony S. Stein, Monzr M. Al Malki, Flavia Pichiorri, Nadia Carlesso, Ya‐Huei Kuo, Guido Marcucci, Ling Li, Jie Jin

**Affiliations:** ^1^ Department of Hematology the First Affiliated Hospital School of Medicine, Zhejiang University Hangzhou Zhejiang PR China; ^2^ Hematological Malignancies Translational Science Gehr Family Center for Leukemia Research City of Hope Medical Center and Beckman Research Institute Duarte California USA; ^3^ Zhejiang Provincial Key Lab of Hematopoietic Malignancy Zhejiang University Hangzhou Zhejiang PR China; ^4^ Zhejiang Laboratory for Systems & Precision Medicine Zhejiang University Medical Center Hangzhou Zhejiang PR China

**Keywords:** CpG‐antimiR‐126, HSPCs, miR‐126, myelodysplastic syndrome, transformation

## Abstract

**Background:**

Myelodysplastic syndrome (MDS) arises from a rare population of aberrant hematopoietic stem and progenitor cells (HSPCs). These cells are relatively quiescent and therefore treatment resistant. Understanding mechanisms underlying their maintenance is critical for effective MDS treatment.

**Methods:**

We evaluated microRNA‐126 (miR‐126) levels in MDS patients’ sample and in a NUP98‐HOXD13 (NHD13) murine MDS model along with their normal controls and defined its role in MDS HSPCs’ maintenance by inhibiting miR‐126 expression in vitro and in vivo. Identification of miR‐126 effectors was conducted using biotinylated miR‐126 pulldown coupled with transcriptome analysis. We also tested the therapeutic activity of our anti‐miR‐126 oligodeoxynucleotide (miRisten) in human MDS xenografts and murine MDS models.

**Results:**

miR‐126 levels were higher in bone marrow mononuclear cells from MDS patients and NHD13 mice relative to their respective normal controls (*P* < 0.001). Genetic deletion of miR‐126 in NHD13 mice decreased quiescence and self‐renewal capacity of MDS HSPCs, and alleviated MDS symptoms of NHD13 mice. Ex vivo exposure to miRisten increased cell cycling, reduced colony‐forming capacity, and enhanced apoptosis in human MDS HSPCs, but spared normal human HSPCs. In vivo miRisten administration partially reversed pancytopenia in NHD13 mice and blocked the leukemic transformation (combination group vs DAC group, P < 0.0001). Mechanistically, we identified the non‐coding RNA PTTG3P as a novel miR‐126 target. Lower PTTG3P levels were associated with a shorter overall survival in MDS patients.

**Conclusions:**

MiR‐126 plays crucial roles in MDS HSPC maintenance. Therapeutic targeting of miR‐126 is a potentially novel approach in MDS.

## Graphical abstract

1. MiR‐126 levels are higher in stem/progenitor cells from MDS patients relative to normal counterparts.
2. MiR‐126 KO inhibits MDS stem/progenitor cell maintenance.
3. In vivo administration of a miR‐126 inhibitor in combination with decitabine partially reverses pancytopenia in a murine MDS model and decreases the risk of leukemic transformation.
4. The non‐coding RNA PTTG3P is a downstream target of miR‐126 in MDS cells.

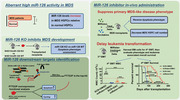

## 1 BACKGROUND

Myelodysplastic syndrome (MDS), is a heterogeneous group of clonal hematopoietic disorders, characterized by blood cytopenia, bone marrow (BM) failure, and a variable risk of transformation into acute myeloid leukemia (AML).^1^ MDS is most prevalent in older adults aged ≥65 years. Currently, approximately 15,000 new MDS cases are diagnosed annually in the U.S., with a 5‐year survival of 38%.^2^

It is broadly accepted that MDS arises from a clonal hematopoietic stem and progenitor cells (HSPCs),^3^, ^4^, ^5^, ^6^ which carry acquired genetic and epigenetic alteration and are enriched in the CD34^+^ subset. These cells eventually become dominant in the BM, thereby contributing to dysplastic cell progenies that fail to properly differentiate into functional mature hematopoietic cells. Therapeutic options for MDS patients, which are prognostically classified according to International Prognostic Scoring System (IPSS) risk stratification, are relatively few. Hypomethylating agents (HMA), such as decitabine (DAC) or azacitidine (AZA), are commonly used in high‐risk MDS and may provide a temporary therapeutic benefit, but they only modestly improve survival.^7^ Allogeneic hematopoietic stem cell transplantation (allo‐HSCT) is the only curative approach for MDS patients. However, patients’ comorbidities, treatment‐related morbidities and mortality, and risk of post‐transplant relapse somewhat limit the applicability of this approach.^8^ Thus, novel, non‐toxic, and more effective therapies are needed to improve the currently disappointing outcomes for MDS patients. To this end, it is critical to understand mechanisms by which MDS HSPCs maintain their activity and then devise therapeutic approaches to eliminate them.

MicroRNAs (miRNAs) are short non‐coding RNAs that hybridize to target messenger RNAs and downregulate target gene expression. Aberrantly expressed miRNAs contribute to the pathogenesis of hematological malignancies including MDS^9^, ^10^ and are reportedly associated with poor clinical outcome of leukemia cases.^11^, ^12^, ^13^ Previous studies have demonstrated a unique role for miR‐126 in maintaining quiescence of leukemia stem cells (LSCs) and enhancing their activity.^12^, ^13^ Thus, we hypothesized here that miR‐126 also contributes to the pathogenesis of MDS, enhancing the clonal hematopoietic activity of HSPCs, and therefore, it may represent a novel therapeutic target in this disease. To this end, we showed that miR‐126 levels were higher in MDS patients compared to normal healthy controls. Downregulation of miR‐126 promoted human MDS CD34^+^ cells into the cell cycle and sensitized them to DAC treatment. Furthermore, miR‐126 inhibition reversed the dysplastic phenotype observed in the NHD13^+^ mouse, a murine MDS model, and decreased engraftment of primary human MDS CD34^+^ cells in immunodeficient mice.

## 2 METHODS

### 2.1 Samples

Bone marrow (BM) samples from healthy donors (n = 10) and MDS patients (n = 52) were obtained from the First Affiliated Hospital, School of Medicine, Zhejiang University for miR‐126 and PTTG3P expression measurement and clinical outcome evaluation (patients’ characteristics, Table S1). Mobilized peripheral blood (PB) from healthy donors (n = 4) and BM samples from MDS patients (n = 10) were obtained at City of Hope National Medical Center (COHNMC) through COH IRB#18067 for further experiments (patients’ characteristics, Table S2). Sample acquisition and analysis were approved by the Institutional Review Boards at Zhejiang University or COHNMC in accordance with the Helsinki Declaration.

### 2.2 Cell culture

MDS‐L was gifted by Dr. Kaoru Tohyama.^14^ MDS‐L cells and Human CD34^+^ cells were cultured as reported previously.^14^, ^15^

### 2.3 Flow cytometric analysis and cell sorting

Cells were analyzed using a Fortessa flow cytometer (BD). Human CD34^+^CD38^+^, CD34^+^CD38^–^ cells and mouse L^–^S^–^K^–^, L^–^S^+^K^–^, L^–^S^–^K^+^, MEP, CMP, GMP, and LSK were isolated by flow cytometry sorting using a FACS Fusion flow cytometer (BD).

### 2.4 Animal studies

Immune competent NHD13 mice (C57BL/6J genetic background, CD45.2) were purchased from Jackson Laboratory. NHD13 mice were mated with conditional miR‐126^f/f^/Mx1‐Cre (immune competence, C57BL/6J genetic background, CD45.2) to obtain NHD13/miR‐126f/f/Mx1‐Cre mice(NHD13/miR‐126 KO mice or NHD13/miR‐126 WT mice).^12^ miR‐126 deletion was induced by polyinosinic‐polycytidylic acid (PIPC) (7 doses, 12 μg/g every other day, intraperitoneal). NHD13/ miR‐126^f/f^/Mx1‐Cre mice cells (CD45.2) were transplanted to CD45.1 B6 mice (Charles River, Wilmington, MA, USA) to track donor CD45.2 murine MDS cells after transplantation. NHD13 mice cells were injected into CD45.1 B6 mice to obtain MDS murine model. MDS‐L cell line or patient MDS CD34^+^ cells were injected into NSG‐SGM3 mice (The Jackson Laboratory, Bar Harbor, ME, USA) to obtain MDS CDX and PDX model respectively. Mouse care and experimental procedures were performed following protocols approved by the COHNMC Institutional Animal Care and Use Committee.

### 2.5 Statistics

Statistical analyses were done by Prism GraphPad (version 8.0, La Jolla, CA). Results are showed as mean ± SEM. Statistical significance was analyzed through Student's t‐test. Kaplan‐Meier survival curves were applied to depict the overall survival, and log‐rank tests were used to estimate the significance of survival. A two‐sided P‐value ˂0.05 was considered significant (Legend: *P < 0.05, **P < 0.01, ***P < 0.001, ****P < 0.0001).

Detailed methods are described in Supporting Information Materials and Methods.

## 3 RESULTS

### 3.1 miR‐126 levels increase in MDS HSPCs relative to normal HSPCs

**FIGURE 1:**
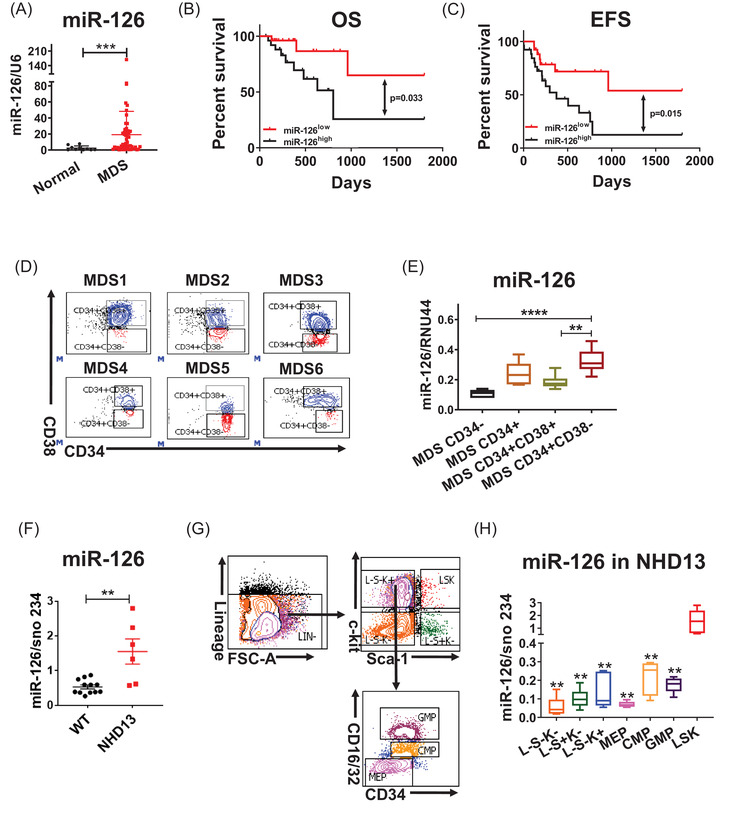
miR‐126 levels increase in MDS HSPCs relative to normal HSPCs. (A) Relative miR‐126 expression in BM mononuclear cells (MNCs) from a cohort of MDS patients (n = 52) and healthy donors (normal; n = 10). (B and C) Overall survival (OS) (B) and event‐free survival (EFS) (C) in MDS patients expressing high and low miR‐126 levels. (F) MiR‐126 expression levels in CD34+ cells from MDS patients and healthy donors. (D and E) Gating strategy for isolation of CD34^−^, CD34^+^, CD34^+^CD38^+^, and CD34^+^CD38^−^ subpopulations (D) and relative miR‐126 expression in those subsets from MDS patients (n = 6) (E). F.Relative miR‐126 expression in BM MNCs from a cohort of normal mice and NUP19/HOXD13 (NHD13) mice. G‐H. NHD13 BM cells were first selected using lineage‐negative population enrichment magnetic beads, then cells of subpopulations were sorted by flow cytometry using a FACS Fusion flow cytometer. Gating strategy used to isolate L^–^S^–^K^–^(Lineage^–^Sca‐1^–^c‐Kit^–^), L^–^S^+^K^–^(Lineage^–^Sca‐1^+^c‐Kit^–^), L^–^S^–^K^+^(Lineage^–^Sca‐1^–^c‐Kit^+^), LSK(Lineage^–^Sca‐1^+^c‐Kit^+^), granulocyte/monocyte progenitors (GMP)(Lineage^–^Sca‐1^–^c‐Kit^+^CD34^+^CD16/32^high+^), common myeloid progenitors (CMP) (Lineage^–^Sca‐1^–^c‐Kit^+^CD34^+^CD16/32^low+^), megakaryocyte erythrocyte progenitors (MEPs) (Lineage^–^Sca‐1^–^c‐Kit^+^CD34^–^CD16/32^–^)(G) as well as miR‐126 levels in different populations of NHD13 mice (H).Results shown represent mean ± SEM. **P < 0.01, ***P < 0.001, ****P < 0.0001; by two‐tailed, paired student's t test. The log‐rank test was used to assess significant differences between survival curves

First, we measured miR‐126 expression levels by qRT‐PCR in BM mononuclear cells (MNCs) from a cohort of MDS patients (n = 52; Table S1) and healthy donors (n = 10); miR‐126 levels were higher in BM MNCs from MDS patients compared to normal counterpart from healthy donors (Figure 1A; *P* = 0.0002). We then subdivided our cohort of MDS patients according to WHO classification, into the following subsets: unclassifiable (MDS‐U), single lineage dysplasia (SLD), multilineage dysplasia (MLD), refractory anemia with excess of blasts 1 (RAEB1), or refractory anemia with excess of blasts 2 (RAEB2). miR‐126 levels were modestly increased in the RAEB‐2 subtype relative to MLD, SLD, or MDS‐U (Figure S1A). We did not observe any significant differences in miR‐126 levels when patients were classified according to the International Prognostic Scoring System (IPSS) (Figure S1B). miR‐126 levels in older patients (> 60 yrs) were slightly increased relative to those seen in younger patients (≤60 yrs) (Figure S1C). We then dichotomized the patient cohort into two groups: higher and lover expressers using the median miR‐126 levels at diagnosis as a cutoff. Higher expressers had shorter overall survival (OS; *P* = 0.033) and event‐free survival (EFS; *P* = 0.015) than patients with lower expression (Figure 1B,C). We also found that miR‐126 levels were higher in MDS CD34^+^ than in CD34^+^ cells from healthy donors (Figure S1D). Of note, within the MDS cell subpopulations, miR‐126 levels were significantly higher in CD34^+^CD38^–^ cells relative to other subsets (Figure 1D,E). These results were recapitulated in healthy MNC^12^ and the NHD13 knock‐in mouse.^16^ An NHD13 fusion gene that initially was identified in a treatment‐related MDS (t‐MDS) patient drives the myeloid dysplasia disease phenotype in mice within 4 months. This model meets all diagnostic criteria for murine myeloid dysplasia disease and recapitulates human MDS^14^, typically with cytopenia (Figure S1E‐I) and BM dysplasia (Figure S1J). We observed miR‐126 levels to be higher in Lin^–^Sca1^+^c‐Kit^+^ (LSK) cells from 20 weeks old NHD13^+^ mice compared with age‐matched normal mice (Figure 1F); we also observed higher miR‐126 levels within more primitive cells [i.e., Lin^–^Sca‐1^+^c‐kit^+^ (LSK) versus LK] (Figure 1G‐H).

### 3.2 miR‐126 KO perturbs MDS maintenance in vivo

**FIGURE 2:**
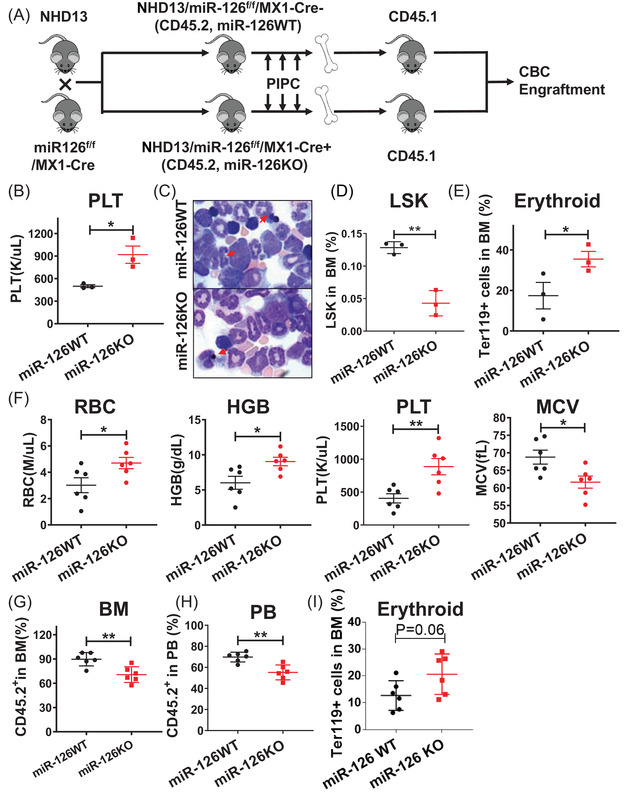
miR‐126 KO perturbs MDS maintenance in vivo. (A) Breeding scheme used to generate NHD13/miR‐126 KO animals. B‐E. PLT counts (B), BM smears(red arrows, dysplasia cells) (C), BM LSK (D) and Ter119^+^ mature erythroid cells (E) in NHD13/miR‐126 KO (n = 3) and NHD13/miR‐126 WT mice (n = 3). F‐I. RBC count, HGB level, PLT count and MCV(F), CD45.2^+^ NHD13^+^ chimerism in BM (G) and PB (H), and the percentage of BM Ter119^+^(I) in secondary recipients receiving with equal numbers of CD45.2^+^ BM cells from either miR‐126 WT or miR‐126 KO NHD13^+^ transgenic mice, at 16 weeks post‐bone marrow transplantation (BMT). Results shown represent mean ± SEM. *P < 0.05, **P < 0.01; by two‐tailed, paired Student's *t*‐test

To assess the role of miR‐126 in the MDS pathogenesis, we then generated NHD13/miR126 KO animals (NHD13/miR‐126^f/f^/Mx1‐Cre) by crossing conditional miR‐126^f/f^/Mx1‐Cre (miR‐126 KO) mice with NHD13 mice (Figure 2A). miR‐126 deletion was induced by intraperitoneal injection with polyinosinic‐polycytidylic acid (PIPC) (12 μg/g every other day, a total of 7 doses). Sixteen weeks after the first PIPC injection, miR‐126 levels in BM cells from NHD13 miR‐126 KO were decreased by 67% compare with NHD13 miR‐126 WT (Figure S2A), similar to findings reported in our previous publication, which decreased miR‐126 levels in BM cells by 60%.^12^ NHD13/miR‐126KO mice had higher platelet (PLT) counts (Figure 2B) and reduced BM dysplasia (Figure 2C) and LSK cells (Figure 2D) and increased Ter119^+^ erythroid cells (Figure 2E) compared to NHD13/miR‐126WT mice. The decreased LSK cell numbers in NHD13/miR‐126KO mice compared with NHD13/miR‐126WT mice likely resulted from miR‐126 KO‐induced apoptosis (Figure S2B‐C).

To evaluate the effects of miR‐126 KO on MDS‐initiation capacity, we then transplanted congenic recipients with equal numbers of CD45.2^+^ BM cells from either miR‐126 WT or KO NHD13^+^ donor mice along with BM cells from WT mice (CD45.1^+^) (9:1). Mice transplanted with NHD13/miR‐126 KO cells had significantly higher RBC, hemoglobin (HGB), and PLT counts and lower mean corpuscular volume (MCV) than those transplanted with NHD13/miR‐126 WT controls by week 16 (Figure 2F). MiR‐126 KO recipients also carried a significantly reduced CD45.2^+^ NHD13^+^ chimerism in BM and peripheral blood (PB) (Figure 2G‐H), suggesting impairment in MDS HSPC self‐renewal capacity. We also observed increased numbers of Ter119^+^ erythroid cells (Figure 2I) in recipients with NHD13/miR‐126 KO relative to those of NHD13/miR‐126 WT.

### 3.3 Inhibition of miR‐126increases human MDS cell cycling and sensitizes cells to DAC treatment

**FIGURE 3:**
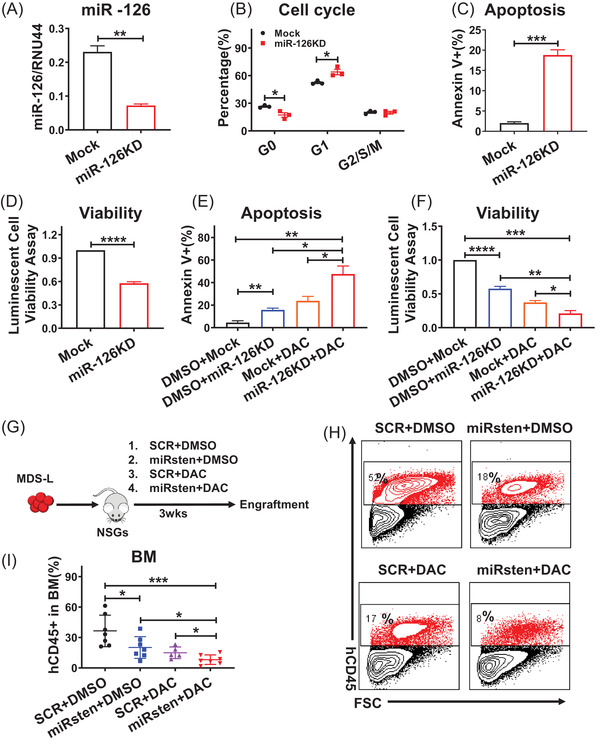
Inhibition of miR‐126 increases MDS‐L cell cycling and sensitizes cells to DAC treatment. A‐D. Relative miR‐126 expression level (A), cell cycling, as indicated by Ki‐67 staining (B), apoptosis, as indicated by Annexin V staining (C), and viability, as measured by luminescence assays (D) in MDS‐L miR‐126KD and control (Mock) cells. E‐F. Apoptosis, as indicated by Annexin V staining (E), and viability (F), based on luminescence assays, in MDS‐L miR‐126KD and control (Mock) cells exposed to DAC. (G) Experimental design was used to establish MDS‐L‐derived xenografts. NSGS mice were transplanted with MDS‐L cells to generate a cohort of mice. After engraftment was confirmed, mice were treated with SCR, miRisten (10 mg/kg/day, once a day), DAC (0.5 mg/kg/day, 3 times per week), or amiRisten/DAC combination for three wks. BM, PB, and SP cells were then harvested and analyzed. H: Representative flow cytometry plots of human CD45 cells (hCD45) in BM. I: BM hCD45 cell engraftment in mice bearing xenografted MDS‐L cells *in‐vivo* administrated with SCR, miRisten, DAC, or the miRisten/DAC combination (n = 5‐7 per group). Results shown represent mean± SEM. *P < 0.05, **P < 0.01, ***P < 0.001,****P < 0.0001; NS: not significant; by two‐tailed, paired Student's *t*‐test

To determine the functional significance of miR‐126 in human MDS, we then knocked down (KD) miR‐126 with an anti‐miR‐126 lentiviral vector (miR‐126 KD) coexpressing GFP in human MDS derived line MDS‐L. miR‐126 KD promoted cell cycle entry, enhanced apoptosis, and reduced cell viability relative to controls (Figure 3A‐D). Since DAC is one of the front‐line treatments for high‐risk MDS patients,^17^ we then asked whether miR‐126 KD could enhance the DAC activity on MDS cells. To this end, we exposed MDS‐L miR‐126KD and control (Mock) cells to DAC for 72 h. Notably, miR‐126 KD significantly sensitized MDS cells to DAC treatment, as evidenced by increased apoptosis and further reduced cell viability relative to DAC treatment alone (Figure 3E‐F).

We recently reported efficient and cell‐specific delivery of oligonucleotide therapeutics (ONTs) to target miR‐126 in vivo (see Zhang et al, 2018, Nat Med). Briefly, we linked a miR126a‐specific antisense oligodeoxynucleotide (ODN) to a cytosine–guanine dinucleotide (CpG) ODN, a ligand for the intracellular protein Toll‐like receptor 9 (TLR9). To enable systemic administration, we chemically modified the inhibitor (CpG–miR‐126i, miRisten) to resist serum nucleases. MiRisten can be taken up by hematopoietic cells efficiently (Figure S3A).^12^ We incubated MDS‐L cells with CpG–miR‐126i conjugated with Cy3. Flow cytometric analysis at 24 hrs after treatment showed that CpG–miR‐126i–Cy3 was taken up by nearly all the cells (Figure S3A). Ex vivo exposure to miRisten led to miR‐126 downregulation in 80% murine MDS HSPCs cells at 24 h (Fig. S3B). To further explore the activity of miRisten alone and in combination with DAC on MDS cell growth in vivo, we established a cohort of NSGS mice xenografted with MDS‐L, and then divided them into four groups with either CpG–scrRNA (SCR), miRisten (10 mg/kg/day, once per day), DAC (0.5 mg/kg/day, 3 times per weeks), or a combination of miRisten/DAC for three weeks. Treated mice were analyzed for BM human cell engraftment (Figure 3G). miRisten or DAC single treatment alone inhibited MDS cell engraftment in NSGS recipients comparing to control group, respectively. Moreover, the miRisten/DAC combination more potently decreased MDS‐L engraftment in BM, PB, and spleen (SP) than did either drug treatment alone (n = 5‐7 per group; Figure 3H‐I, S3C‐D).

**FIGURE 4:**
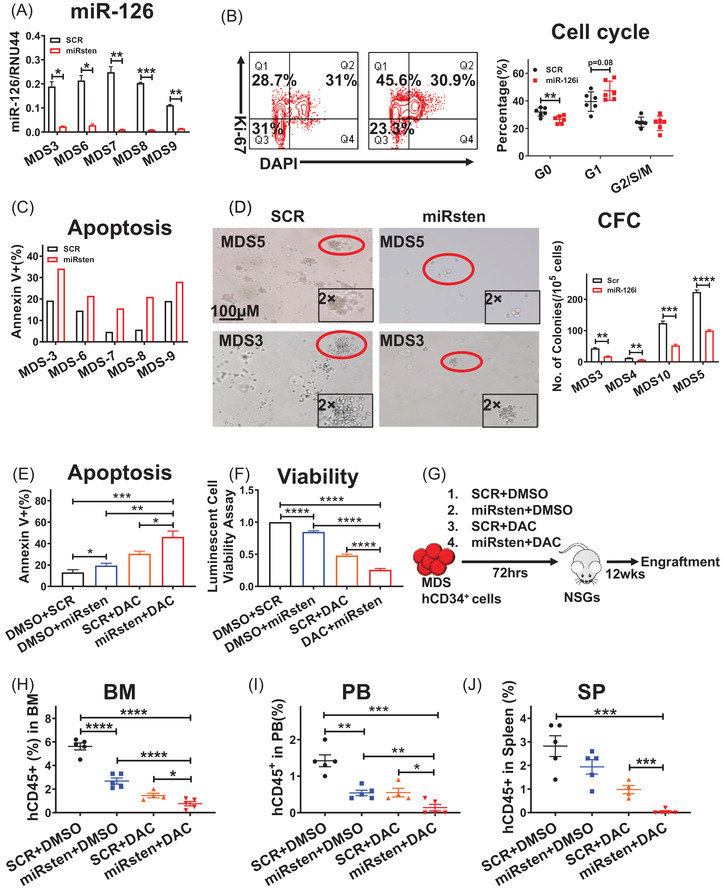
Inhibition of miR‐126 increases human MDS cell cycling and sensitizes cells to DAC treatment. A‐D. Relative miR‐126 expression levels (A), cell cycling, as indicated by Ki‐67 staining (B), apoptosis, as indicated by Annexin V staining, (C) and colony‐forming cell (CFC) (D) in primary MDS CD34^+^ cells exposed to miRisten or SCR. (E and F) Apoptosis, as indicated by Annexin V staining (E), and viability, as measured by luminescence assays (F), in primary MDS CD34^+^ cells exposed 72 h to SCR, miRisten, DAC or a miRisten/DAC combination. G. Experimental design used to establish MDS patient‐derived xenografts. NSGS mice were transplanted with MDS CD34^+^ cells after cells were treated ex vivo with SCR, miRisten, DAC, or the miRisten/DAC combination for 72 h. BM, PB, and SP cells were then harvested and analyzed. H‐J: hCD45^+^ cell engraftment in BM (H), PB (I), and SP (J) in NSGS mice treated with SCR, single drug, or the miRisten/DAC combination at 12 weeks post‐BMT (n = 4‐5 per group). Results shown represent mean± SEM. *P < 0.05, **P < 0.01, ***P < 0.001,****P < 0.0001; NS, not significant; by two‐tailed, paired Student's *t*‐test

We also evaluated miRisten effects on primary MDS CD34^+^ cells. Notably,miRisten treatment promoted cell cycle entry, induced apoptosis, and reduced colony formation capacity of primary MDS CD34^+^ cells compared to SCR treatment (Figure 4A‐D). In MDS cells exposed to the SCR control, miRisten (500 nM), DAC (1 μM), or the miRisten plus DAC, we observed that the combination treatment significantly enhanced apoptosis and reduced MDS cell viability relative to either drug treatment alone (Figure 4E‐F). To determine if there was a synergistic effect between miRisten and DAC, MDS‐L cells were exposed to SCR, miRisten, DAC or a miRisten/DAC combination for 72 h. The result showed combination index calculated by CalcuSyn software was less than 1, which indicated miRisten and DAC have a synergistic effect in MDSL cells (Figure S3E‐F). To assess miRisten effects on primary human MDS cell in vivo repopulating capacity, we treated MDS CD34^+^ cells *ex vivo* for 72 h with SCR control, miRisten, DAC, or the miRisten/DAC combination and transplanted treated cells into NSGS mice (Figure 4G). Twelve weeks later, mice that had received miRisten‐ or DAC‐treated MDS cells showed significantly reduced human MDS cell engraftment comparing to those of controls. Importantly, the combination treatment more potently inhibited MDS cell engraftment in BM, PB, and SP relative to mice that had received cells treated with either drug alone or the SCR control (Figure 4H‐J).

### 3.4 miRisten treatment does not impair normal HSPCs function

We next asked if miRisten treatment impairs normal human HSPC function. To this end, we treated CD34^+^ cells from mobilized normal peripheral blood with miRisten (500 nM) or SCR for 72 h *ex vivo* (n = 3). miRisten treatment had modest effects on either apoptosis (Figure S4A) or viability of normal cells (Figure S4B). To assess miRisten effect on normal HSPC in vivo repopulating capacity, we treated normal CD34^+^ cells *ex vivo* with miRisten or SCR for 72 h and then transplanted cells into NSGS mice (Figure S4C). Twelve weeks later, we observed no significant difference of human cell engraftment in NSGS mice xenografted with cells treated with miRisten or SCR, indicating miRisten treatment could spare normal HSPCs activity while the treatment potently inhibits MDS cells growth in vivo (Figure S4D‐F). Moreover, we also treated normal B6 mice with miRisten (10 mg/kg, i.v., 3 weeks) and observed no significant impact on hematologic output (not shown).

### 3.5 miRisten treatment ablates MDS maintenance

**FIGURE 5:**
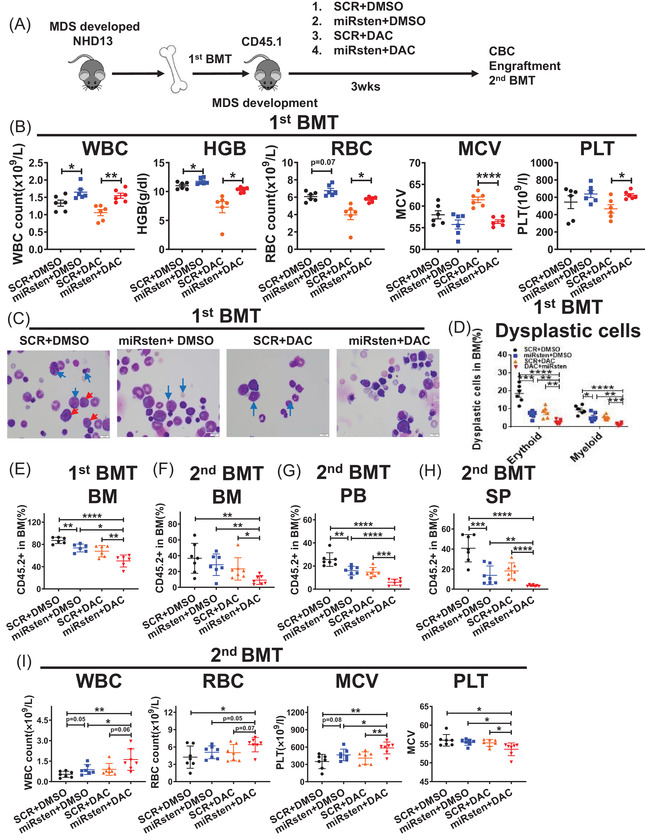
miRisten treatment ablates MDS HSPCs in NHD13 transgenic mice. (A) CD45.2^+^ BM cells from donor NHD13^+^ mice were transplanted into CD45.1 preconditioned congenic recipients. Recipient animals then underwent treatment with SCR, miRisten (10 mg/kg/day), DAC (0.5 mg/kg, 3 times/week), or the miRisten/DAC combination for 3 weeks (n = 6 per group). BM, PB, and SP were harvested and analyzed, and BM cells from each group were transplanted into secondary CD45.1 recipients. (B) WBC count, HGB levels, RBC counts, MCV, and PLT counts in each group after treatment. (C) BM smears from each treatment group (red arrow, blasts; blue arrows, dysplasia cells). (D) Percentage of dysplasia cells in BM in each treatment group. (E) Percentage of CD45.2, engraftment in BM in each treatment group. (F and G) Percentage of CD45.2 engraftment in BM (F), PB (G) and SP (H) and WBC count, HGB levels, RBC count, MCV and PLT count (I) in secondary transplants receiving BM cells from single drug‐, combination‐, or SCR‐treated donors at 16 weeks post‐BMT (n = 7 per group). Results shown represent mean ± SEM. *P < 0.05, **P < 0.01, ***P < 0.001,****P < 0.0001; by two‐tailed, paired Student's *t*‐test

We next evaluated the effects of in vivo administration of miRisten by transplanting CD45.2^+^ BM MNCs from primary donor mice developing MDS together with WT support cells (CD45.1^+^) at the ratio of 9:1 into sublethally‐irradiated congenic recipients. When the transplanted mice showed first signs of MDS, as evidenced by abnormal complete blood cell (CBC) counts around 20 weeks post‐BMT, we started treatment with SCR, miRisten (10 mg/kg/day), DAC (0.5 mg/kg, 3 times/week) or the combination of miRisten and DAC for 3 weeks (n = 6, each group) (Figure 5A). miRisten treatment led to approximately 70% miR‐126 reduction in NHD13 BM cells (Figure S5A), and increased WBC, HGB, RBC, and PLT counts relative to controls (Figure 5B). Dysplastic cells in recipient's BM were also decreased following miRisten treatment (Figure 5C‐D). Importantly, engraftment of CD45.2^+^ MDS cells was largely decreased in BM after drug treatment (Figure 5E). Interestingly, unlike DAC as a single agent, mice receiving the combination treatment had less pancytopenia, along with a decreased BM dysplastic cell number and donor MDS engraftment. (Figure 5B‐E). Combination treatment more potently increased the number of erythrocytes relative to DAC single treatment (Fig. S5B). In addition, miRisten treatment significantly reduced number of CD45.2^+^ LSK cells. While we observed no changes in cell cycling, treatment with the miRisten increased apoptosis in the CD45.2^+^ LSK population in BM from NHD13 transplants (Figure S5C‐D).

In secondary transplantation experiments, 2 × 10^6^ BM cells from control or drug‐treated primary transplants were transplanted into a cohort of CD45.1 recipients. Sixteen weeks post‐BMT, we observed that the recipients of BM cells from miRisten‐treated donors showed reduced CD45.2^+^ chimerism in BM, PB, and SP and decreased dysplasia cell percentage compared with recipients of BM cells from control donors (Figure 5F‐G, S5E‐F). We also observed modest changes in CBC counts in mice receiving miRisten alone treated BM cells (Figure 5I). Notably, mice receiving BM cells from the combination‐treated mice showed the most reduced CD45.2^+^ cell engraftment and a significant reverse of cytopenia evidenced by increased WBC, RBC, and PLT counts relative to recipients of BM cells from donors treated with either drug alone or vehicle control (Figure 5F‐I). The number of dysplastic cells from the combination group was further reduced relative to those of miRisten alone treatment (Figure. S5E‐F).

### 3.6 MiRisten treatment blocks NHD13^+^leukemia transformation

The recipients transplanted with NHD13^+^ MDS BM cells not only displayed the critical MDS features such as pancytopenias, BM cells dysplasia, but also exhibited a risk of AML transformation. To assess whether miR‐126 inhibition could block the transformation potential of NHD13^+^ MDS, we performed serial transplantation using CD45.2^+^ BM MNCs from primary NHD13^+^ donor mice. Leukemia transformation was observed in the quaternary transplants, and was characterized with high WBC, and higher blasts in BM (> 20%) (not shown)as defined.^14,16^

**FIGURE 6:**
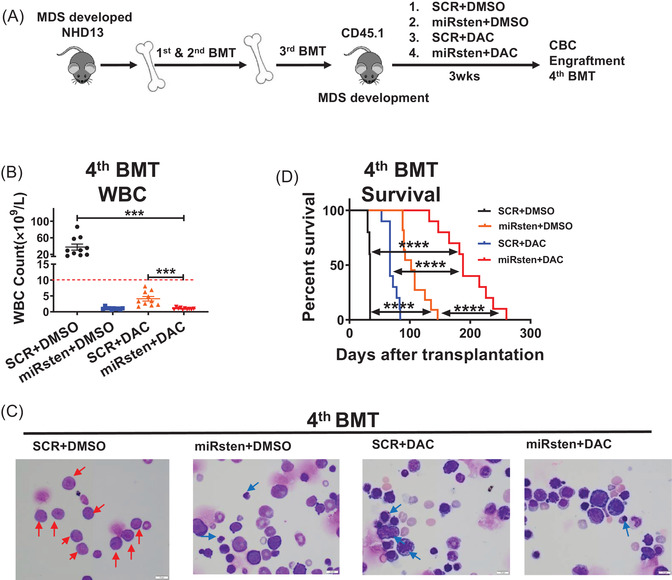
miRisten treatment blocks leukemia transformation in a murine MDS model. (A) After a serial transplant, we have set up a cohort of tertiary transplant animals. Then we treated the transplants with SCR, miRisten (10 mg/kg/day), DAC (0.5 mg/kg, 3 times/week), or a combination of miRisten and DAC for 3 weeks (n = 5‐6 per group) after robust engraftment, BM, PB, and SP were harvested and analyzed, then BM cells in each group were transplanted into next CD45.1 recipients (quaternary transplants) to monitor leukemia transformation. (B‐D) WBC counts (B), BM smears (red arrows, blasts; blue arrow, dysplasia cells) (C), and survival curves (D) derived from quaternary transplant mice that received BM cells from donors that had undergone indicated treatments. Results shown represent mean ± SEM. ***P < 0.001,****P < 0.0001; NS: not significant; by two‐tailed, paired Student's *t*‐test. The log‐rank test was used to assess significant differences between survival curves

To evaluate the effects of miRisten on leukemia transformation, we then treated tertiary transplanted NHD13^+^ mice with SCR, miRisten (10 mg/kg/day), DAC (0.5 mg/kg, 3 times/week), or the miRisten/DAC combination for 3 weeks (Figure 6A). We showed that miRisten treatment alleviated MDS symptoms, miRisten, DAC, or their combination treatment reduced donor cell engraftment and blast cell percentage in the BM of the third transplant recipients (Figure S6A‐D). Of note, these mice were in an MDS state as evidenced by the low WBC counts and BM blast cell percentage (< 20%). Then we harvested an equal number of BM cells from mice and transplanted them into a cohort of quaternary recipients that we monitored for leukemia transformation and survival. Relative to recipient mice receiving cells from miRisten, DAC, or combination‐treated third transplants, recipient mice receiving BM cells from control third transplants developed leukemia in a short period with high WBC and BM blast percentage (> 20%) (Figure 6B,C). Notably, miRisten treatment seemed more potent than single DAC treatment on delaying leukemia transformation as evidenced by delayed WBC expansion (>10,000 cells/μl)and longer survival of fourth recipients transplanted with miRisten treated BM relative to those of DAC treated BM (median survival: combination 188 days versus DAC 67 days, *P* < 0.0001) (Figure 6B‐D). Moreover, we found significantly extended survival of fourth recipients transplanted with miRisten treated BM relative to those of vehicle control‐treated BM (median survival: miRisten 102 days vs vehicle control 34 days, *P* < 0.0001) (Figure 6D), while in control and miRisten treatment group, all mice died of leukemia, as evidenced by high WBC counts and blasts (Figure 6B‐C). In DAC treatment group, six of 10 mice died of leukemia, as evidenced by high WBC counts and blasts; three mice died of infection, as evidenced by skin ulceration and weight loss in combination with low WBCs/PLTs; one mouse died of weight loss combined with low WBCs/PLTs. In the combination treatment group, 6 out of 10 mice died of leukemia; three mice died of infection, as evidenced by skin ulceration, weight loss, and low WBCs/ PLTs, and one mouse died of weight loss and low WBCs/PLTs without no sign of infection (Table S3).

### 3.7 Identification of miR‐126 downstream targets in MDS

**FIGURE 7:**
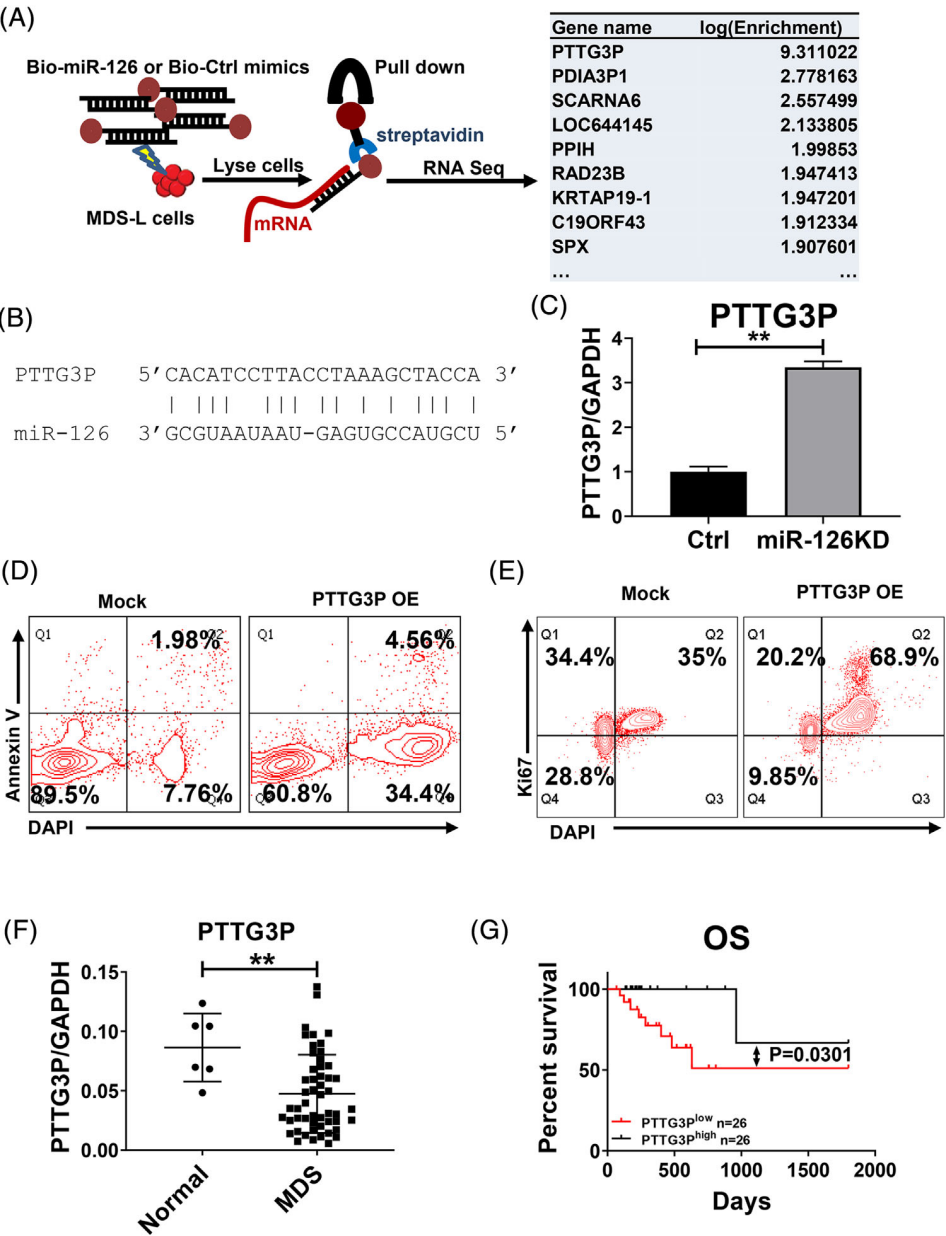
Identification of miR‐126 downstream targets in MDS. (A) Schematic showing the Biotin‐miR‐126 pull‐down assay. (B) Predicted miR‐126 binding sites in PTTG3P mRNA. (C) PTTG3P expression in MDS‐L cells transduced with Mock or anti‐miR‐126 lentiviral vector. GAPDH served to normalize expression. (D) Representative flow cytometry plots assessing apoptosis (based on Annexin V staining) of MDS‐L cells transduced with mock or lentivirus vector overexpressing (OE) PTTG3P. (E) Representative flow cytometry plots assessing cell cycle of MDS‐L cells transduced with Mock or a lentiviral PTTG3P OE vector. (F) PTTG3P expression in BM MNCs from normal healthy donor (n = 6) vs MDS (n = 52) cells. GAPDH served to normalize expression. (G) OS of MDS patients with low or high PTTG3P expression. Results shown represent mean ± SEM. **P < 0.01; by two‐tailed, paired Student's *t*‐test. The log‐rank test was used to assess significant differences between survival curves

To understand the mechanisms underlying miR‐126 function in MDS pathogenesis, it is necessary to delineate the targets of miR‐126 in MDS cells. To this end, we first electroporated MDS‐L cells with a 3′‐biotinylated miR‐126 mimic or scrambled control, followed by RNA capture using streptavidin beads, which specifically capture RNAs bound to the exogenous biotinylated miRNA mimic. Through RNA sequencing of the pulled‐down transcripts (Figure 7A), we identified 96 genes whose mRNA were markedly enriched by the miR‐126 mimic relative to scramble control pull‐down group (fold‐change > 2, *P* < 0.05) (Table S4). Interestingly, we found a novel long non‐coding RNA, Pituitary Tumor‐Transforming 3, Pseudogene (PTTG3P), as the top enriched mRNA in this assay (*P* < 0.01). Notably, miR‐126 binding sites in PTTG3P were shown (Figure 7B). MDS‐L cells transduced with anti‐miR‐126 lentiviral vector indeed increased PTTG3P mRNA levels relative to those transduced with control vector (Figure 7C). PTTG3P expression is reportedly associated with increased cell proliferation^17‐21^. When we ectopically expressed PTTG3P in MDS‐L cells, we also observed increased cell cycling and enhanced MDS‐L apoptosis (Figure 7D‐E), strongly suggesting that PTTG3P counteracts miR‐126 function in MDS cells. Notably, PTTG3P KD decreased MDS‐L apoptosis in miR‐126 KD cells, while PTTG3P KD alone did not alter MDS‐L apoptosis (Figure S7A). To confirm PTTG3P direct binding of miR‐126, we synthesized 3′‐biotinylated miR‐126 WT mimics, some single site mutated miR‐126 mimics at some potential PTTG3P binding sites (G3A, C7T, G12A, A14G, G20A, or G22A), all potential binding sites mutated mimics (the “All‐mut” miR‐126 mimic), and scramble oligos, then transduced these oligos into MDS‐L cells through electroporation and cultured for 24 h. RNA capture from cell lysate was performed using streptavidin beads. PTTG3P was enriched by the non‐mutant miR‐126 mimic but not by the 3′‐biotinylated scramble oligo or the All‐mut construct, while single mutations only modestly affected miR‐126 binding to PTTG3P (Figure S7B). Importantly, PTTG3P levels in MDS cells were significantly lower than those seen in normal controls (Figure 7F), and patients with higher than the median level of PTTG3P showed longer OS than those with lower PTTG3P levels (Figure 7G). In our cohort of MDS patients, we observed higher PTTG3P levels in healthy CD34^+^ cells compared to those from MDS counterparts (Figure S7C). Our analysis of an existing dataset (GSE19429) revealed that PTTG3P levels in the CD34^+^ subset of MDS‐RAEB (36.85 vs 47.11, *P* = 0.0003) were lower than those in normal counterparts (Figure S7D‐E). Importantly, we also observed an inverse correlation between PTTG3P and miR‐126 levels in MDS CD34^+^ cells within an individual case (Figure S7F, *R* = 0.37, *P* = 0.04).

## 4 DISCUSSION

MDS HPSCs which are characterized by aberrant self‐renewal capacity persist even after conventional therapy, giving rise to aberrant progenies.^3^ Allogeneic stem cell transplantation is the only curative treatment for MDS patients, however, its efficacy is limited by associated toxicity, with a long‐term survival rate of ∼30%.^24^ Recent transcriptome analysis based on a large cohort of MDS cases revealed that a hematopoietic stem cell gene signature is positively associated with poor prognosis, independent of blast percentage,^25^ suggesting that targeting these primitive MDS cells could lead to a potential cure. Herein, we report that miR‐126 levels are aberrantly higher in MDS BM HSPCs relative to more mature subset; the treatment of miR‐126 inhibitor reversed dysplasia of murine MDS mice and blocked human MDS cell engraftment in vivo, supporting further clinical investigation of whether targeting miR‐126 would represent a therapeutic approach against MDS.

To the best of our knowledge, the role of miR‐126 in MDS has not yet been characterized. Similar to our previous study of chronic myeloid leukemia (CML), we found higher miR‐126 levels in more primitive subsets (CD34^+^CD38^–^) of MDS as compared to mature subsets (CD34^+^CD38^+^). To assess the effects of miR‐126 function on MDS development in vivo, we generated NHD13/miR126 KO animals for loss‐of‐function studies. Analysis of this mouse showed that miR‐126 maintains MDS HSPC self‐renewal capacity, given that miR‐126 KO decreased dysplasia phenotypes of fully developed disease as well as the MDS initiation. Mechanistically, miR‐126 depletion promotes cell cycle entry likely by exhausting the aberrant HSPC pool, an observation consistent with our previous reports, including our observations of leukemia stem cells..^12^, ^26^, ^27^ Our previous study indeed revealed that enforced miR‐126 expression drove cells into a quiescent stage. To test whether miR‐126 is a druggable target, we recently developed an efficient and cell‐specific method to deliver oligonucleotide therapeutics (ONTs) and used one of those constructs (miRisten) to target miR‐126. Interestingly, miRisten treatment of normal B6 mice (10 mg/kg, i.v., 3 weeks) did not exhibit any detectable hematologic toxicity but rather increased hematopoietic output, as evidenced by higher hemoglobin levels. In the context of murine MDS, miRisten combined with DAC treatment enhanced DAC induced ablation of MDS clones relative to DAC treatment alone, indicating that miR‐126 could serve as a potential therapeutic target against MDS.

Human MDS is characterized by a high risk of progression to acute myeloid leukemia (AML)^1^. NHD13 mouse used here represents an excellent model in which to assess MDS progression to leukemia: approximately one‐third of NHD13 mice with MDS develop leukemia, most commonly AML, which mimics disease progression seen in MDS patients.^28^, ^29^ Given unique aspects of MDS pathogenesis, using an NHD13 transplant model, we found that miR‐126 inhibition blocked leukemia transformation by disrupting homeostasis of the MDS HSPC pool. Also, in serial transplantation assays, we found that quaternary recipients receiving cells from miRisten/DAC combination‐treated tertiary transplants indeed showed longer survival without blast expansion in the BM.

Lechman et al. has reported that miR‐126 targets PI3K/AKT/mTOR signaling partly through CDK3, preserving LSC quiescence and promoting chemotherapy resistance in AML.^17^ Thus, we hypothesize that miR‐126 direct target could be context‐dependent, we searched for miR‐126 targets in the MDS‐L cells. We previously reported that in CML cells miR‐126 mainly targets SPRED1, thereby regulating p‐ERK/p‐BCL2 signaling and functioning in cell survival.^12^ Herein, following miR‐126 transfection/pulldown assay combined with RNA‐seq, we identified transcripts specifically targeted by ectopically expressed miR‐126 and among them, the non‐coding gene PTTG3P was the top‐ranked candidate. Given that PTTG3P overexpression induces cell cycling and apoptosis, it is possible that this non‐coding gene could be a major miR‐126 target that plays a role in MDS. Moreover, MDS patients with lower than median PTTG3P levels showed shorter OS than patients with higher PTTG3P levels. Future studies should address the function of PTTG3P in MDS pathogenesis.

## 5 CONCLUSIONS

We have first shown that higher miR‐126 levels are associated with MDS pathogenesis. miR‐126 deletion decreased MDS HSPC cell growth and self‐renewal capacity, thereby compromising the pool of MDS HSPCs. Targeting miR‐126 by miR‐126 inhibitor not only blocked MDS development but seemingly reduced the risk of MDS further transformation, thereby supporting this as a potentially novel therapeutic approach for MDS patients.

## CONFLICT OF INTEREST

The authors declare no potential conflict of interest.

## Supporting information



Supporting Information

## Data Availability

The data that supports the findings of this study are available in the supplementary material of this article.
