# Supplementary material for: Targeting miR‐126 disrupts maintenance of myelodysplastic syndrome stem and progenitor cells
Source: Clin Transl Med. 2021 Oct 14;11(10):e610. doi: 10.1002/ctm2.610 (PMC8516361; doi:10.1002/ctm2.610)
Supplement: Supplementary file 1 — Supporting Information [file CTM2-11-e610-s001.DOCX]

**Targeting miR-126 disrupts maintenance of myelodysplastic syndrome stem and progenitor cells**

Huafeng Wang, MD^1,2,3,4*^, Jie Sun, PhD^2*^, Bin Zhang, PhD^2*^, Dandan Zhao, MD^2*^, Hongyan Tong, MD^1,3^, Herman Wu, BS^2^, Xia Li, PhD^1^, Yingwan Luo, MD^1^, Dan Dong, PhD^2^, Yiyi Yao, BS^1,3^, Tinisha McDonald, BS^2^, Anthony S. Stein, MD^2^, Monzr M. Al Malki, MD^2^, Flavia Pichiorri, PhD^2^, Nadia Carlesso, PhD^2^, Ya-Huei Kuo, PhD^2^, Guido Marcucci, MD^2#^, Ling Li, PhD^2#^, Jie Jin, MD，PhD^1,3#^

^1^Department of Hematology, the First Affiliated Hospital, College of Medicine, Zhejiang University, Hangzhou, Zhejiang, PR China

^2^Hematological Malignancies Translational Science, Gehr Family Center for Leukemia Research, City of Hope Medical Center and Beckman Research Institute, Duarte, CA, USA

^3^Zhejiang Provincial Key Lab of Hematopoietic Malignancy, Zhejiang University, Hangzhou, Zhejiang, PR China

^4^Zhejiang Laboratory for Systems & Precision Medicine, Zhejiang University Medical Center, Hangzhou, Zhejiang, PR China

^*^These four authors contributed equally to this work.

Table S1: Characteristic of MDS patients.

| Patient No. | Subtype (2016 WHO) | Age | Gender | IPSS score |
| --- | --- | --- | --- | --- |
| 1 | SLD | F | 59 | 0.5 |
| 2 | MLD | F | 60 | 1.5 |
| 3 | SLD | M | 27 | 0.0 |
| 4 | RAEB1 | F | 61 | 1.5 |
| 5 | RAEB2 | M | 61 | 2.0 |
| 6 | MLD | F | 54 | 0.5 |
| 7 | MLD | M | 75 | 0.5 |
| 8 | MDS-U | M | 42 | 0.0 |
| 9 | MLD | F | 22 | 0.5 |
| 10 | RAEB2 | M | 56 | 2.5 |
| 11 | MLD | F | 44 | 0.5 |
| 12 | RAEB2 | F | 67 | 2.5 |
| 13 | RAEB1 | F | 25 | 1.0 |
| 14 | MDS-U | M | 69 | 0.0 |
| 15 | MLD | M | 37 | 0.5 |
| 16 | MLD | M | 56 | 0.5 |
| 17 | RAEB1 | M | 53 | 1.5 |
| 18 | RAEB1 | M | 75 | 1.5 |
| 19 | RAEB1 | M | 62 | 2.0 |
| 20 | RAEB2 | F | 51 | 2.5 |
| 21 | SLD | M | 57 | 0.0 |
| 22 | RAEB2 | F | 59 | 2.0 |
| 23 | MLD | M | 65 | 0.5 |
| 24 | MLD | M | 57 | 1.0 |
| 25 | RAEB1 | F | 61 | 1.0 |
| 26 | MLD | F | 34 | 1.0 |
| 27 | MLD | M | 57 | 1.0 |
| 28 | SLD | F | 66 | 0.5 |
| 29 | SLD | M | 43 | 0.0 |
| 30 | RAEB2 | F | 47 | 1.5 |
| 31 | RAEB2 | F | 73 | 1.0 |
| 32 | RAEB1 | M | 66 | 2.5 |
| 33 | MLD | M | 64 | 0.5 |
| 34 | SLD | M | 73 | 0.5 |
| 35 | RAEB2 | M | 76 | 2.5 |
| 36 | RAEB2 | F | 70 | 1.5 |
| 37 | MDS-U | F | 65 | 0.5 |
| 38 | MLD | F | 51 | 1.0 |
| 39 | SLD | M | 59 | 1.5 |
| 40 | RAEB2 | M | 35 | 2.0 |
| 41 | RAEB1 | M | 63 | 1.0 |
| 42 | RAEB1 | M | 30 | 2.0 |
| 43 | RAEB2 | F | 56 | 1.0 |
| 44 | RAEB2 | M | 29 | 1.5 |
| 45 | RAEB2 | M | 47 | 1.5 |
| 46 | RAEB1 | M | 59 | 1.0 |
| 47 | RAEB2 | M | 56 | 1.5 |
| 48 | MLD | M | 71 | 0.5 |
| 49 | RAEB2 | M | 77 | 2.0 |
| 50 | RAEB1 | M | 58 | 1.5 |
| 51 | RAEB2 | F | 51 | 1.0 |
| 52 | RAEB2 | M | 51 | 2.0 |

Table S2: Characteristics of patients for experiments.

| Patient No. | Diagnosis (2016 WHO) | Age | Gender | Cytogentic |
| --- | --- | --- | --- | --- |
| 1 | RAEBI | 67 | F | del(7) |
| 2 | RAEB-I | 44 | M | t(6;16), del(14) |
| 3 | RAEBII | 78 | M | 47, XY, +8[20] Trisomy 8 95.7% by FISH |
| 4 | SLD | 60 | M | Trisomy 8 |
| 5 | RAEBII | 65 | F | NA |
| 6 | MLD | 42 | M | del(7) |
| 7 | RAEBII | 66 | M | trisomy 8 |
| 8 | MLD | 57 | M | Normal |
| 9 | SLD | 63 | F | del(5q) |
| 10 | RAEBII | 63 | F | Normal cytogenetics |

Table S3: WBC and PLT in four treated group mice before death.

|  |  | | M1 | M2 | M3 | M4 | M5 | M6 | M7 | M8 | M9 | M10 |
| --- | --- | --- | --- | --- | --- | --- | --- | --- | --- | --- | --- | --- |
| Control | | WBC(×10^9^/L) | 17.91 | 57.59 | 29.92 | 29.12 | 86.68 | 22.67 | 61.29 | 16.34 | 19.24 | 35.46 |
|  | | PLT(×10^9^/L) | 152 | 130 | 246 | 109 | 95 | 162 | 229 | 577 | 208 | 202 |
| miRisten | | WBC(×10^9^/L) | 28.63 | 39.72 | 76.53 | 58.32 | 19.21 | 25.78 | 46.69 | 109.2 | 38.5 | 47.92 |
|  | | PLT(×10^9^/L) | 77 | 106 | 132 | 108 | 85 | 176 | 96 | 468 | 356 | 303 |
| DAC | | WBC(×10^9^/L) | 38.63 | 18.72 | 26.35 | 72.86 | 38.7 | 53.42 | 0.13 | 0.09 | 0.67 | 0.93 |
|  | | PLT(×10^9^/L) | 146 | 126 | 456 | 126 | 75 | 103 | 195 | 106 | 156 | 224 |
| Combination | | WBC(×10^9^/L) | 32.78 | 30.56 | 56.88 | 20.85 | 19.21 | 48.52 | 1.32 | 0.78 | 0.16 | 0.36 |
|  | | PLT(×10^9^/L) | 217 | 158 | 95 | 116 | 90 | 87 | 108 | 118 | 285 | 368 |

M:mouse; WBC: white blood cell; PLT: platelet.

Table S4：miR-126 pulldown markedly enriched 96 genes (more than 2 folds)

| genes | log(Enrichment) |
| --- | --- |
| PTTG3P | 9.311022 |
| PDIA3P1 | 2.778163 |
| SCARNA6 | 2.557499 |
| LOC644145 | 2.133805 |
| PPIH | 1.99853 |
| RAD23B | 1.947413 |
| KRTAP19-1 | 1.947201 |
| C19ORF43 | 1.912334 |
| SPX | 1.907601 |
| SPRR2F | 1.896347 |
| SENP8 | 1.884384 |
| SDHAF3 | 1.870887 |
| LOC100506100 | 1.841505 |
| HIST1H2BE | 1.819007 |
| RPL37A | 1.801491 |
| SWI5 | 1.800373 |
| MALAT1 | 1.685331 |
| TCTEX1D2 | 1.676911 |
| ZCCHC12 | 1.629404 |
| LAMTOR3 | 1.576943 |
| RILP | 1.570768 |
| PSMB9 | 1.569644 |
| CAV2 | 1.5628 |
| CAMK2N1 | 1.508813 |
| OR7E14P | 1.488549 |
| NMRK1 | 1.457921 |
| ZNF124 | 1.456268 |
| FAM221A | 1.450081 |
| RAB7A | 1.449693 |
| MRPS14 | 1.447561 |
| FAM86HP | 1.438814 |
| TMEM163 | 1.434659 |
| LSM7 | 1.404743 |
| STPG1 | 1.338267 |
| SOD1 | 1.335061 |
| APOM | 1.331034 |
| METTL1 | 1.315425 |
| GMNN | 1.306082 |
| RNF130 | 1.302632 |
| SCAMP2 | 1.2968 |
| PQLC3 | 1.288914 |
| CENPN | 1.283953 |
| GEMIN7 | 1.276253 |
| LYRM9 | 1.259119 |
| RPS16 | 1.254338 |
| LOC100507547 | 1.248252 |
| HDX | 1.244566 |
| ADPRM | 1.215649 |
| GRAMD3 | 1.214387 |
| TP53I3 | 1.208054 |
| GSC | 1.20669 |
| RIPK2 | 1.200004 |
| GLO1 | 1.189476 |
| RNASE4 | 1.185942 |
| TMEM225B | 1.174294 |
| NDUFC1 | 1.169597 |
| ZNF396 | 1.168602 |
| LSM3 | 1.166557 |
| RPL35 | 1.159183 |
| GATA5 | 1.159168 |
| HIST1H1T | 1.15881 |
| TPM1 | 1.15497 |
| C19ORF38 | 1.13463 |
| HMX3 | 1.126411 |
| UFSP1 | 1.12598 |
| YPEL3 | 1.120038 |
| IFIT1 | 1.114278 |
| DDIT4 | 1.104578 |
| IGFBP6 | 1.101073 |
| UCK1 | 1.095678 |
| SDSL | 1.091176 |
| MTX1 | 1.086125 |
| RPS24 | 1.084835 |
| LXN | 1.076778 |
| SNCB | 1.074019 |
| DGCR6 | 1.068993 |
| PLEKHA8P1 | 1.068549 |
| C1ORF56 | 1.068377 |
| SYCE2 | 1.060112 |
| HOXB9 | 1.058313 |
| PRPF38B | 1.0574 |
| HIST1H3E | 1.054918 |
| EFNA3 | 1.054004 |
| CDK8 | 1.038515 |
| PRTFDC1 | 1.036755 |
| DUSP2 | 1.036503 |
| MPZL3 | 1.028349 |
| RNF181 | 1.025059 |
| TMEM53 | 1.019196 |
| ZCCHC18 | 1.018841 |
| RUSC1 | 1.014858 |
| BTG2 | 1.014119 |
| HIST2H2BF | 1.011651 |
| NDUFB4 | 1.009683 |
| GNG5 | 1.00774 |
| BEND5 | 1.003132 |

**Figure S1: *miR-126 levels increase in MDS HSPCs relative to normal HSPCs****.* A-C. MiR-126 expression level in MDS-U, MDS-SLD, MDS-MLD, MDS-RAEB-1 and MDS-RAEB-2 subtype (A), in low-, intermediate-1(int-1)- or intermediate-2(int-2)- or high-risk groups according to IPSS score (B) and in old patients (>60 yrs) and young patients (≤60 yrs) (C). D. MiR-126 expression levels in CD34+ cells from MDS patients and healthy donors. E-J. White blood cell (WBC) count (E), red blood cell count (RBC) (F), hemoglobin (HGB) levels (G), mean corpuscular volume (MCV) (H), platelet (PLT) (I) counts and BM smear (blue arrow, dysplasia cells) (J) in WT (n=15) and NHD13 (n=17) mice. Results shown represent mean± s.e.m. *P < 0.05, **P < 0.01, ****P < 0.0001, NS: not significant; by two-tailed, paired student’s t test. The log-rank test was used to assess significant differences between survival curves.

**Figure S2:** ***miR-126 KO perturbs MDS maintenance in vivo.*** A. MiR-126 level in BM cell of NHD13 miR-126flox/flox/Mx1-cre+ (Mx1+) and NHD13 miR-126flox/flox/Mx1-cre- (Mx1-) mice at 16 weeks after PI:PC injection (n=3 independent samples). B-C. LSK counts and apoptosis indicated by annexin V in primary NHD13/miR-126KO mice and NHD13/miR-126KO mice. Results shown represent mean± s.e.m. *P<0.05, **P < 0.01, ***P < 0.001; by two-tailed, paired student’s t test.

**Figure S3: *miRisten uptake and its effect on MDS-L cells in vitro and in vivo*.** A-B. Flow cytometry analysis for Cy-3 (A) and relative miR-126 expression (B) 24 hours after MDS-L cells were exposed to Scr-Cy3 or miRisten-Cy3 (500 nM). C-D. hCD45 engraftment in PB (C) and SP (D) in mice bearing MDS-L cells *in vivo* administrated with SCR, miRisten, DAC or a combination of miRisten and DAC (n=5-7 per group). E. Viability, as measured by luminescence assays, in MDSL cells exposed to SCR, miRisten, DAC or a miRisten/DAC combination as indicated concentration for 72 hours. F. Combination index was calculated by CalcuSyn software. Results shown represent mean± s.e.m. *P < 0.05, **P < 0.01, by two-tailed, paired student’s t test.

**Figure S4: *miRisten treatment has no effect on long-term in vivo repopulating capacity of normal HSPCs****.* A-B. Apoptosis as indicated by Annexin V staining (A) or viability, as measured by luminescence assays (B) of normal human CD34^+^ cells (n=3) exposed to miRisten or SCR. C. Schematic showing experimental design used to establish human CD34^+^ cell-derived xenografts. D-F. hCD45^+^ cell engraftment in BM (D), PB (E) and SP (F) in NSG mice treated with SCR or miRisten at 12 wks post-BMT (n=6-7 per group). Results shown represent mean± s.e.m. NS: not significant based on two-tailed, paired student’s t test.

**Figure S5: *miRisten treatment ablates MDS HSPCs in NHD13 transgenic mice.*** Dysplastic CD45.2+ BM cells from donor NHD13+ mice were transplanted into CD45.1 preconditioned congenic recipients, which were then treated with SCR, miRisten (10mg/kg/day), DAC (0.5mg/kg, 3 times/ week) or a miRisten/DAC combination for 3 wks (n=6 per group). BM, PB and SP were harvested and analyzed, and BM cells in each group transplanted into secondary CD45.1 recipients. A. BM miR-126 levels after 3 weeks in vivo miRisten treatment (10mg/kg, iv, per day, n=3). B. Percentage of donor derived Ter119+ cell engrafted in the recipient BM after indicated treatments (n=6 per group). LSK counts (C) and apoptosis (D) indicated by annexin V in SCR, miRisten, DAC or a miRisten/DAC combination treated NHD13 transplanted mice. E. BM smear (blue arrow, dysplasia cells) and percentage of dysplasia cells in BM (F) of secondary transplants receiving BM cells from indicated donors at 16 wks post-BMT (n=7 per group). Results shown represent mean± s.e.m. *P < 0.05, **P < 0.01, ***P < 0.001, ****P < 0.0001; by two-tailed, paired student’s t test.

***Figure S6: miRisten treatment blocks leukemia transformation in a murine MDS model.***

After a serial transplant, we have set up a cohort of tertiary transplant animals. Then we treated the transplants with SCR, miRisten (10mg/kg/day), DAC (0.5mg/kg, 3 times/ week) or combination of miRisten and DAC for 3 wks (n=5-6 per group) after robust engraftment, BM, PB and SP were harvested and analyzed, then BM cells in each group were transplanted into next CD45.1 recipients (quaternary transplants) to monitor leukemia transformation. A: WBC count, RBC count, HGB levels, MCV and PLT in each group of tertiary transplants after treatment. B-C. BM CD45.2 (B) and CD45.2 LSK (C) cell engraftment in each group of tertiary transplants after treatment. D. Representative BM smears (red arrow, blasts; blue arrow, dysplasia cells) from each group of tertiary transplants after treatment. Results shown represent mean± s.e.m. *P < 0.05, **P < 0.01; by two-tailed, paired student’s t test.

***Figure S7: Identification of miR-126 downstream targets in MDS.*** A. PTTG3P shRNA or control vector was transduced to wild type MDS-L cells, or the ones stably expressed anti-miR-126 by lentivirus transduction. Cell apoptosis was evaluated by Annexin V staining. B. MDS-L cells were transduced with 3-biotinylated miR-126 mimic, mutated miR-126 mimic or scrambled control through electroporation and cultured for 24 hours. RNA capture from cell lysate was performed using streptavidin beads enrichment of PTTG3P was evaluated by qPCR. C. PTTG3P expression levels in CD34+ cells of healthy donors and MDS patients. D-E. PTTG3P expression levels in CD34+ cells of healthy donors, total MDS patients(D), RA/RARS and RAEB-1/2 subtype(E data from gene expression dataset (GSE19429). . F. Correlation between PTTG3P levels and miR-126 levels of CD34+ cells within individual patients. Results shown represent mean± s.e.m. *P < 0.05, **P<0.01, ***P < 0.001, ****P < 0.0001; NS: not significant by two-tailed, paired student’s t test.

**Supplemental materials and methods**

**Sample, miRisten, Scramble and DAC**

Mononuclear cells (MNCs) were isolated using Ficoll separation and CD34^+^ cells were selected through magnetic beads (Miltenyi Biotech, Cologne, Germany). miRisten and Scramble were made as described previously^1^. DAC was purchased from Sigma (cat A3656).

**Flow cytometric analysis**

Cells were analyzed using Fortessa flow cytometer (BD). Human CD34+CD38+, CD34+CD38– cells and mouse L-S-K-, L-S+K-, L-S-K+, MEP, CMP, GMP and LSK were isolated by flow cytometry sorting using FACS Fusion flow cytometer (BD).The following anti-mouse antibodies were used: biotinylated lineage antibodies (all from eBioscience, San Diego, CA, USA) against: Flt3 (clone A2F10, cat 13-1351-85), Ter119 (clone TER-119, cat 13-5921-85), NK1.1 (clone PK136, cat 13-5941-85), CD11b (clone M1/70, cat 13-0112-85), Gr-1 (clone RB6-8C5, cat 13-5931-85), IgM (clone eB121-15F9, cat 13-5790-85), CD19 (clone eBio1D3 (1D3), cat 13-0193-85), B220 (clone RA3-6B2, cat 13-0452-85), CD3 (clone 17A2, cat 13-0031-85), CD4 (clone GK1.5, cat 13-0041-85) and CD8 (clone 53-6.7, cat 13-0083-85), other mouse antibodies against: CD117 (APC-eflu780, clone ACK2, cat 47-1172-82, eBioscience), Sca-1 (PE–Cy7, clone D7, cat 25-5981-82, eBioscience), CD45.1(PE, clone A20, cat 553776, BD Biosciences), Ter119 (APC-eflu 780, clone TER-119, cat 47-5921-82 ), B220 (FITC, clone RA3-6B2, cat 11-0452-82), CD45.2 (APC, clone 104, cat 558702, BD Biosciences) and anti-streptavidin (FITC, cat 11-4317-87, eBioscience), CD16/32(PE-Cy7, clone 93, cat 25-0161-82) and CD34(eflu 660, clone RAM34, cat 50-0341-82).The following human antibodies were used: human antibodies against CD45 (FITC, clone 2D1, cat 11-9459-42, Thermo Fisher Scientific, Waltham, MA, USA ; APC, clone HI30, cat 561864, BD Biosciences ), CD34 (APC, clone 581, cat 555824, BD Biosciences; PE-Cy7,clone 581,cat 560710, BD Biosciences), CD38 (PE, clone HB7, cat 12-0388-41, eBioscience) and Annexin V (PE, 559763, BD Biosciences; APC, 640941, BioLegend, San Diego, CA, USA).

**Apoptosis，viability colony-forming assays and cell cycle**

To measure apoptosis, cells were stained with Annexin V and 4, 6-diamidino-2-phenylindole (DAPI) (BD Biosciences) and analyzed by flow cytometry (BD Biosciences).Viability was determined with Cell Titer-Glo® Luminescent Cell Viability Assay (Promage, Madison, WI, USA). For CFC assay, MDS CD34+ cells were cultured with SCR or miRisten (500nM, 72 hours), then plated in methylcellulose progenitor culture medium [1:1 mixed by "Base" Methylcellulose Medium for Human Cells Base Media (04100, Stem Cell) and CFC medium (60% FBS, β-mercaptoethanol10μM, rhEPO 10U/ml,  rhIL-3 10ng/ml, rhSCF 10ng/ml, rhGM-CSF 40ng/ml, rhG-CSF 40ng/ml)] (all cytokines from Pepro Tech US, β-mercaptoethanol from Sigma, St. Louis, MO, USA).2 Then colony-forming unit-granulocyte and macrophage cells were counted after 14 days. Cell cycle was measured by using the Mouse Anti-Ki-67 Set (BD Biosciences, cat 556027) following the manufactory’s protocol.

**Morphological Analysis**

Morphological analysis of mice cells as described previously3. Briefly, cells were spread onto slides, stained and evaluated based on morphology for dysplastic phenotypes. Cytospins from BM, SP samples were prepared by diluting 2x10^5^ cells in 100 mL PBS and spinning them on glass microscope slides. Blood smears were prepared by pipetting 5 mL of fresh blood mixed with EDTA on a glass microscope slide and spreading it by pulling the edge of another microscope slide over the surface of the first slide starting from the blood drop. Differential staining was performed as the protocol provided by the Wright-Giemsa Stain kit. Briefly, cells on the slides were fixed in absolute methanol for 1 minute and stained in Wright-Giemsa Stain Solution for 1 minute, then smears were rinsed in Phosphate Buffer pH 6.6 solution for 5 minutes. Slides were examined under a microscope after washing with running water. Three BM cytospin slides were scored from each individual animal. Six animals of each group were evaluated; 100 cells were scored on each slide.

**Engraftment of human cells in immunodeficient mice**

MDS-L cells were transplanted (1x10^6^ cells/mouse) via tail vein injection into sublethallyirradiated (280 cGy) 8-week-old NSG-SGM3 mice. After engraftment was confirmed, the mice were treated for 3 wks with SCR, miRisten (10mg/kg/day, once per day), DAC (0.5mg/kg/day, 3 times per weeks) and combination of miRisten plus DAC (n=5-7 per group). Engraftment of human CD45^+^ (hCD45^+^) cells in BM, PB or SP was analyzed by flow cytometry. For primary MDS CD34^+^ cells engraftment, after ex vivo treatment with SCR, miRisten (500nM), DAC (1 µM) and the combination for 72 hours, CD34^+^ MDS cells (2×10^6^ cells/mouse) were transplanted into sublethally-irradiated (280 cGy) 8-week-old female NSGS mice (n=4-5 per group). Twelve wks later, engraftment of hCD45^+^ cells in BM, PB or SP was analyzed by flow cytometry. For normal CD34^+^ cells engraftment, after ex vivo treatment with SCR and miRisten (500nM) for 72 hours, CD34+ normal cells were transplanted into sublethally-irradiated (280 cGy) 8-week-old female NSG mice (n=6-7 per group), engraftment of hCD45^+^ cells in BM, PB or SP was analyzed by flow cytometry at 12 wks after transplantation.

***In vivo* treatment of transgenic NHD13 mice**

After the NHD13 mice developed MDS (about 4 months), BM cells (CD45.2) were collected and transplanted (2×10^6^ cells/mouse) via tail vein injection into 6-8-week-old irradiated (600 cGy) CD45.1 congenic recipient mice. After confirming MDS development, mice were treated with SCR, miRisten (10mg/kg/day, once per day), DAC (0.5mg/kg/day, 3 times per weeks) and combination of miRisten plus DAC for 3 wks (n=6 per group), then PB, BM, and SP cells were harvested and analyzed for MDS burden. CBC (Drew Hemavet 950FS) were analyzed. The number of total nucleated cells, CD45.2^+^ cells as well as mature and progenitor cell was determined by flow cytometry. Then, BM cells from the treated mice were pooled and transplanted into irradiated CD45.1 recipient mice (600cGy) (2×10^6^ cells/mouse). Sixteen weeks later, CBC and engraftment of donor-derived cells in PB，BM and SP cells was evaluated. For leukemia transformation in this NHD13 mice, we found leukemia transformation during the quaternary transplant. A cohort of tertiary transplants were treated as indicated four groups, analysis and do the 4th transplantation.

**miR-126 pull-down assay**

Biotin miR-126 pull-down assay was performed as described previously4. Briefly, MDS-L were transfected in triplicate with 3′-biotinylated miR-126 mimic or scrambled control through electroporation (Lonza) and cultured for 24 hours. Then the cells washed with PBS and resuspended in lysis buffer with RNase OUT (Invitrogen). The cell lysate was then incubated with yeast tRNA and BSA blocked Streptavidin-coated magnetic beads (Invitrogen) for 4 hours at 4˚C. RNA bound to the beads and the RNA in the primary cell lysate (input) were isolated using Trizol LS reagent (Invitrogen) and were sequenced followed by standard Illumina protocols on the Illumina HiSeq 2500 platform (Illumina). The sequence reads were then mapped to human genome (GRCh37) using STAR (v2.6.0.a). The raw counts of each gene were generated by running HTSeq (v0.6.1p1) against GRCh37 GTF file (Ensembl release 83). The enrichment ratio was calculated using the formula of (miR-126-pulldown counts/Ctrl-pulldown counts)/(miR-126-input counts/Ctrl-input counts).

**Lentiviral transduction**

miRZip™ anti-miR-126 expression lentivectors and its control were purchased from System Biosciences, PTTG3P cDNA was synthesize by Genscript and subcloned to pCDH-EF1α-MCS-IRES-GFP lentivector (System Biosciences). Lentiviruses were produced and used for transduction of MDS-L cells. MDS-L cells were resuspended in culture medium and lentiviral supernatant, supplemented with 1× TransDux virus transduction reagent (System Biosciences,), and centrifuged at 1,500g for 90 min for transduction.

**Real-Time Quantitative PCR analysis**

Total RNA was extracted using the miRNeasy Mini Kit (Qiagen, Valencia, CA, USA). For PTTG3P expression, first-strand cDNA was synthesized using the SuperScript III First-Strand Kit (Life Technologies), and quantitative real time PCR was performed using SYBR Green master mix (Life Technologies) with gene-specific primers. Signals were detected with a QuantStudio 7 Flex Real-Time PCR system (Life Biotechnology). The primers for PTTG3P were 5’-AAACGAAGAACCAGGCATCCTT-3’ and 5’-GGGAGCATCGAATGTTTTGCC-3’. For miR-126 expression, reverse transcription using MultiScribe Reverse Transcriptase and qPCR analysis using Taqman probe to miR-126 (cat 4427975) were performed according to the manufacturer’s protocol. Signals were detected with a QuantStudio 7 Flex Real-Time PCR system (Life Biotechnology). Results are presented as a log2-transformed ratio according to the 2−ΔCt method (ΔCt = Ct of target −Ct of reference).

**Reference:**

1. B. Zhang, L.X.T. Nguyen, L. Li, D. Zhao, B. Kumar, H. et al, Bone marrow niche trafficking of miR-126 controls the self-renewal of leukemia stem cells in chronic myelogenous leukemia. Nat Med. 2018; 24(4):450-462

2. Wang H, Zhao D, Nguyen LX, Wu H, Li L, Dong D, et al. Targeting Plasma Membrane HDM2: A Potentially Novel Therapeutic Approach for Acute Myeloid Leukemia (AML). Leukemia.2020; 34 (1): 75–86.

3. Sun J, He X, Zhu Y, Ding Z, Dong H, Feng Yet al. SIRT1 Activation Disrupts Maintenance of Myelodysplastic Syndrome Stem and Progenitor Cells by Restoring TET2 Function. Cell Stem Cell. 2018;23(3):355-369.e9.

4. A. Lal, M.P. Thomas, G. Altschuler, F. Navarro, E. O'Day, X.L. Li, et al. Hide, and J. Lieberman,

Capture of microRNA-bound mRNAs identifies the tumor suppressor miR-34a as a regulator of growth factor signaling. PLoS Genet. 2011;7(11):e1002363.: PMC3213160..

S1

**Funding Information section**

This work was supported in part by National Natural Science Foundation of China (No.81800146); Key international cooperation projects of the National Natural Science Foundation of China(No.81820108004);Youth Natural Science Foundation of Zhejiang Province, China (LQ18H080001); US National Cancer Institute grants: CA102031 (GM), CA201184 (GM), CA180861 (GM), CA158350 (GM); the Gehr Family Foundation.MDS-L was kindly gifted from Dr. Kaoru Tohyama. Research reported in this publication included work performed in the Animal Resources Center, Analytical Cytometry, Hematopoietic Tissue Bank Coreand Light Microscopy Cores at City of Hope Comprehensive Cancer Center supported by the National Cancer Institute of the National Institutes of Health under award number P30CA33572. The content is solely the responsibility of the authors and does not necessarily represent the official views of the National Institutes of Health. We are grateful to COH Comprehensive Cancer Center and the First Affiliated Hospital, College of Medicine, Zhejiang University, the patients and their physicians for providing primary patient material for this study.

**Aothor-Supplied Data**

Huafeng Wang, MD^1,2,3,4#^, Jie Sun, PhD^1,2,3#^, Bin Zhang, PhD^2#^, Dandan Zhao, MD^2#^, Hongyan Tong, MD^1,3^, Herman Wu, BS^2^, Xia Li, PhD^1^, Yingwan Luo, MD^1^, Dan Dong, PhD^2^, Yiyi Yao, BS^1,3^, Tinisha McDonald, BS^2^, Anthony S. Stein, MD^2^, Monzr M. Al Malki, MD^2^, Flavia Pichiorri, PhD^2^, Nadia Carlesso, PhD^2^, Ya-Huei Kuo, PhD^2^, Guido Marcucci, MD^2*^, Ling Li, PhD^2*^, Jie Jin, MD，PhD^1,3*^

^1^Department of Hematology, the First Affiliated Hospital, School of Medicine, Zhejiang University, Hangzhou, Zhejiang, PR China, 310003

^2^Hematological Malignancies Translational Science, Gehr Family Center for Leukemia Research, City of Hope Medical Center and Beckman Research Institute, Duarte, CA, USA, 91010

^3^Zhejiang Provincial Key Lab of Hematopoietic Malignancy, Zhejiang University, Hangzhou, Zhejiang, PR China, 310003

^4^Zhejiang Laboratory for Systems & Precision Medicine, Zhejiang University Medical Center, Hangzhou, Zhejiang, PR China, 310000

**Correspondence should be addressed to:**

Jie Jin (lead contact), the First Affiliated Hospital, College of Medicine, Zhejiang University, 79 Qingchun Road, Hangzhou, Zhejiang 310003, PR China. Phone: 86-571-87236702; Fax: 86-571-87236702. Email: [jiej0503@zju.edu.cn](mailto:jiej0503@zju.edu.cn)

or Ling Li, City of Hope Medical Center, 1500 E Duarte Road, Duarte, CA 91010, USA. Phone: 626-218-2011; Fax: 626-301-8973. Email: [LingLi@coh.org](mailto:LingLi@coh.org)

or Guido Marcucci, City of Hope Medical Center, 1500 E Duarte Road, Duarte, CA 91010, USA. Phone: 626-218-2705; Fax: 626-301-8973. Email: [gmarcucci@coh.org](mailto:gmarcucci@coh.org)
